# AssemblyQC: a Nextflow pipeline for reproducible reporting of assembly quality

**DOI:** 10.1093/bioinformatics/btae477

**Published:** 2024-07-30

**Authors:** Usman Rashid, Chen Wu, Jason Shiller, Ken Smith, Ross Crowhurst, Marcus Davy, Ting-Hsuan Chen, Ignacio Carvajal, Sarah Bailey, Susan Thomson, Cecilia H Deng

**Affiliations:** Molecular & Digital Breeding, The New Zealand Institute for Plant and Food Research Limited, 1025 Auckland, New Zealand; Molecular & Digital Breeding, The New Zealand Institute for Plant and Food Research Limited, 1025 Auckland, New Zealand; Molecular & Digital Breeding, The New Zealand Institute for Plant and Food Research Limited, 3182 Te Puke, New Zealand; Molecular & Digital Breeding, The New Zealand Institute for Plant and Food Research Limited, 1025 Auckland, New Zealand; Molecular & Digital Breeding, The New Zealand Institute for Plant and Food Research Limited, 1025 Auckland, New Zealand; Molecular & Digital Breeding, The New Zealand Institute for Plant and Food Research Limited, 3182 Te Puke, New Zealand; Molecular & Digital Breeding, The New Zealand Institute for Plant and Food Research Limited, 7608 Lincoln, New Zealand; Molecular & Digital Breeding, The New Zealand Institute for Plant and Food Research Limited, 1025 Auckland, New Zealand; Molecular & Digital Breeding, The New Zealand Institute for Plant and Food Research Limited, 1025 Auckland, New Zealand; Molecular & Digital Breeding, The New Zealand Institute for Plant and Food Research Limited, 7608 Lincoln, New Zealand; Molecular & Digital Breeding, The New Zealand Institute for Plant and Food Research Limited, 1025 Auckland, New Zealand

## Abstract

**Summary:**

Genome assembly projects have grown exponentially due to breakthroughs in sequencing technologies and assembly algorithms. Evaluating the quality of genome assemblies is critical to ensure the reliability of downstream analysis and interpretation. To fulfil this task, we have developed the AssemblyQC pipeline that performs file-format validation, contaminant checking, contiguity measurement, gene- and repeat-space completeness quantification, telomere inspection, taxonomic assignment, synteny alignment, scaffold examination through Hi-C contact-map visualization, and assessments of completeness, consensus quality and phasing through k-mer analysis. It produces a comprehensive HTML report with method descriptions, tables, and visualizations.

**Availability and implementation:**

The pipeline uses Nextflow for workflow orchestration and adheres to the best-practice established by the nf-core community. This pipeline offers a reproducible, scalable, and portable method to assess the quality of genome assemblies—the code is available online at GitHub: https://github.com/Plant-Food-Research-Open/assemblyqc.

## 1 Introduction

During the last 20 years, reference genome assemblies have been generated for over 700 plant species (Sun *et al.* 2022). As of May 2024, the Assembly Database hosted by the National Center for Biotechnology Information (NCBI) returned >2.3 million search results (NCBI 2024). This exponential growth in genome assemblies has been realized by the continuous and substantial decrease in the cost of whole-genome sequencing (NHGRI 2023), coupled with advancements of sequencing technologies and assembly algorithms (Agarwal *et al.* 2020, Dida and Yi 2021). This trend is expected to persist as the pursuit for high quality genomes remains a major goal (Rhie *et al.* 2020b). Moreover, the reduced costs allow an increasingly wider community of research labs to routinely assemble new genomes. Aligned with this, fast and standardized assembly quality assessment has become critical and indispensable.

There are three major aspects of assembly quality: contiguity, completeness and correctness (Wang and Wang 2023). The Earth Biogenome Project tracks an updated list of quality metrics and their minimum values which should be met by genome assemblies (EBP 2024). A plethora of bioinformatics tools are available, each focusing on one specific aspect. To comprehensively assess an assembly, the researcher usually needs to run multiple tools to cover various aspects. This is challenging not only because many tools require manual installation and configuration of correct dependencies, but also because it can be tedious and time-consuming to run different tools separately. Reproducibility of the quality assessment results is an even bigger challenge as the problem is compounded by varied runtime requirements across platforms and frequent version changes. For example, BUSCO (Simão *et al.* 2015, Seppey *et al.* 2019), which has been widely adopted to estimate the gene-space completeness of an assembly had 11 updates within a year (January 2022 to June 2023).

To facilitate a streamlined application of the quality assessment tools in a reproducible manner, GenomeQC was recently released (Manchanda *et al.* 2020) with publicly available source code and a free R/Shiny webapp. The Vertebrate Genomes Project (VGP) assembly pipeline provides wrappers to execute different tools, including running individual quality evaluation tools (Rhie *et al.* 2020b). Incorporating additional tools for a thorough quality assessment as compared to existing pipelines, we have developed AssemblyQC, which adopts the highly portable Nextflow workflow management system in combination with the community-curated nf-core framework (Di Tommaso *et al.* 2017, Ewels *et al.* 2020, Langer *et al.* 2024). AssemblyQC is a unified, fully automated, reproducible tool that can be executed on local machines, high-performance computers, or on the cloud to evaluate the quality of genome and transcriptome assemblies. Quality metrics chosen for report are based on community standards. The pipeline is implemented using nf-core modules, which are reviewed and regularly updated by a large open-source community (Langer *et al.* 2024).

## 2 Materials and methods 

### 2.1 Design

**Figure 1.**
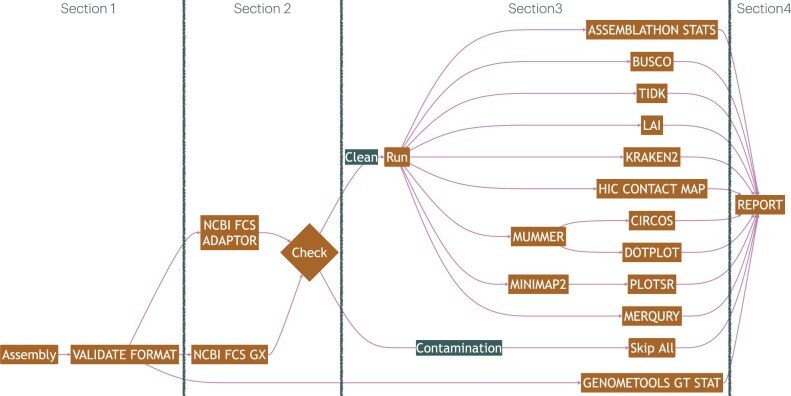
Pipeline flowchart.

The pipeline is designed to evaluate multiple genome or transcriptome assemblies in parallel. The pipeline is divided into four major sections as shown in the flowchart in Fig. 1. Within each section, data are processed in parallel where possible. In Section 1, the pipeline checks the input FASTA and GFF3 annotation files using *py_fasta_validator*, SekQit *rmdup*, and GenomeTools *gt gff3validator* (Gremme *et al.* 2013, Shen *et al.* 2016, Edwards 2019), to ensure integrity of the input files, detect duplicate sequences and prevent failure of subsequent tools. In Section 2, the pipeline uses NCBI’s Foreign Contamination Screen (FCS) and its database to detect contaminants such as adapters and sequences from foreign organisms (Astashyn *et al.* 2023). Assemblies that successfully pass these checks move on to additional quality checks in Section 3. The pipeline can be configured to skip quality checks for assemblies which contain contaminants.

Section 3 executes the remaining quality assessment in parallel for each assembly. For scaffold and contig-level contiguity statistics associated with FASTA sequences, *assemblathon2-analysis* is used (UCDAVIS-Bioinformatics 2012). To break the assembly into contigs, the pipeline uses 100 ‘N’ bases as the default unknown gap size. This parameter can be changed and is quoted in the AssemblyQC final report atop the contig-level statistics. Statistics related to annotations in GFF3 files are computed with GenomeTools *gt stat* tool (Gremme *et al.* 2013). The Benchmarking Universal Single-Copy Orthologs (BUSCO) tool is used for estimating the gene-space completeness of each assembly (Seppey *et al.* 2019). The pipeline can be configured to evaluate each assembly against one or more BUSCO lineages in parallel. The quality of the repeat-space is evaluated with the Long Terminal Repeat (LTR) Assembly Index (LAI) (Ou *et al.* 2018). The *LTR_retriever* workflow consisting of *LTR_FINDER* and *LTRharvest* for *de novo* detection of LTRs (Ellinghaus *et al.* 2008, Ou and Jiang 2018, 2019). The LAI statistic is independent of the BUSCO statistics and can help improve overall assembly contiguity by isolating shortcomings in the repeat-space.

The pipeline can also assess chromosome completeness and correctness through telomere, Hi-C contact-frequency, and synteny visualizations. The Telomere Identification toolKit (tidk) is employed to estimate the presence of telomeres with a specified telomeric motif (Brown *et al.* 2023). To supplement assessment with this user-specified repeat motif, the toolkit also explores the most likely data-driven repeat sequence for each assembly from the assembly sequences. The results for TIDK are sorted in ascending order by sequence length using SeqKit (Shen *et al.* 2016). Presence of the telomeric repeats throughout a sequence often indicates assembly errors, and in many cases, these can be corrected by rearranging fragments in the sequence.

Where Hi-C data are provided, a contact-frequency map is generated by the pipeline and added to the report for visualization. FASTQC and FASTP are used to trim and quality check the Hi-C reads (Andrews 2010, Chen *et al.* 2018). The reads are then mapped to the assembly sequences using Burrow-Wheeler Aligner (BWA) (Heng Li 2013). The mapping quality is checked with *hic_qc.py* (Sullivan 2022), and finally converted into *hic* format through a workflow consisting of *samblaster*, *samtools*, and *run-assembly-visualizer.sh* (Faust and Hall 2014, Dudchenko *et al.* 2017, Danecek *et al.* 2021). The interactive visualization of the Hi-C map is added to the report using the *Juicebox.js* JavaScript library (Robinson *et al.* 2018). Atypical Hi-C contact-frequency patterns such as gaps or nondiagonal contact concentrations can indicate gaps in the assembly or misassignment of contigs to scaffolds.

Two sub-workflows, the pair-wise mode, and the chromosome-level comparison, are implemented in the pipeline to generate synteny plots between input assemblies. The pair-wise workflow creates synteny plots between each combination of input assemblies. Firstly, cross-mapping is performed with MUMmer4 (Marçais *et al.* 2018). The pipeline can be configured to include or exclude many-to-many mappings detected by MUMmer4. The mappings are then plotted with Circos (Krzywinski *et al.* 2009). A dot-plot can also be created which is helpful in identifying inversions (Cabanettes and Klopp 2018). This result makes it easy to visualize gaps and large structural variations (SVs) between assemblies and helps to consistently assign chromosomal numbers to those assemblies. Chromosome-level comparison sub-workflow maps corresponding chromosomes in all the input assemblies with Minimap2 (Li 2018), and generates synteny plots with *plotsr* (Li 2018, Goel and Schneeberger 2022), allowing chromosome-by-chromosome visualization of the key synteny blocks and large SVs from all input. This sub-workflow is helpful for creating a single summary synteny visualization.

Kraken2 is used to assign taxonomic labels to each assembly sequence (Wood *et al.* 2019) and an interactive report is produced with Krona (Ondov *et al.* 2011). This can be useful in detecting cross-domain contamination in the assemblies (Cornet and Baurain 2022).

Merqury is also included in the pipeline to facilitate k-mer analysis. The pipeline supports both mixed-haplotype and diploid assembly assessment with and without the availability of parental reads. In addition to k-mer completeness and consensus quality, Merqury is very useful in evaluating the extent of haplotype phasing (Rhie *et al.* 2020a).

In Section 4 of the pipeline, outputs from various assessment tools are gathered, parsed, and converted into a HyperText Markup Language (HTML) report using Jinja templating language in *Python*. Other than file format validation, the remaining assessment tools are optional and can be bypassed via settings in the configuration file.

### 2.2 Implementation details

The implementation of the pipeline follows the Nextflow good-practice developed by the nf-core community (Ewels *et al.* 2020, Langer *et al.* 2024). It is built with the nf-core pipeline template, and whenever feasible, utilizing the nf-core modules and sub-workflows. The nf-test is used for unit testing, with each module executing a single script or tool. The modules do not rely on installation of dependencies in the system environment; instead, they are implemented using version-locked Bioconda Docker/Singularity/Apptainer containers (Merkel 2014, da Veiga Leprevost *et al.* 2017, Kurtzer *et al.* 2017, Grüning *et al.* 2018) from public repositories including https://quay.io and https://hub.docker.com. Pipeline code, documentation and example reports are available on GitHub: https://plant-food-research-open.github.io/assemblyqc.

## 3 Conclusion

We have created a highly portable and reproducible pipeline for comprehensive assessment of genome and transcriptome assemblies. The pipeline evaluates contiguity, completeness, correctness, and contamination of assemblies in multiple ways with various well adopted bioinformatics tools. The quality assessment results are presented in a shareable HTML report. Furthermore, the actionable insights from the report such as locations of contaminants, locations and abundance of telomeric motifs inside chromosome sequences, presence of gaps or off diagonal contacts in the Hi-C map, and structural variations borne out by the synteny plots, can be used to guide iterative improvement of the assembly.

## Data Availability

Pipeline usage documentation, parameter descriptions, and example outputs are available on GitHub: https://github.com/Plant-Food-Research-Open/assemblyqc/tree/main/docs. A preview report is also hosted online: https://plant-food-research-open.github.io/assemblyqc.
